# Serial Passaging of *Candida albicans* in Systemic Murine Infection Suggests That the Wild Type Strain SC5314 Is Well Adapted to the Murine Kidney

**DOI:** 10.1371/journal.pone.0064482

**Published:** 2013-05-30

**Authors:** Anja Lüttich, Sascha Brunke, Bernhard Hube, Ilse D. Jacobsen

**Affiliations:** 1 Department of Microbial Pathogenicity Mechanisms, Leibniz Institute for Natural Product Research and Infection Biology – Hans Knoell Institute Jena (HKI), Jena, Germany; 2 Center of Sepsis Control and Care, Jena, Germany; 3 Friedrich Schiller University, Jena, Germany; University of Wisconsin Medical School, United States of America

## Abstract

The opportunistic fungal pathogen *Candida albicans* has a remarkable ability to adapt to unfavorable environments by different mechanisms, including microevolution. For example, a previous study has shown that passaging through the murine spleen can cause new phenotypic characteristics. Since the murine kidney is the main target organ in murine *Candida* sepsis and infection of the spleen differs from the kidney in several aspects, we tested whether *C. albicans* SC5314 could evolve to further adapt to infection and persistence within the kidney. Therefore, we performed a long-term serial passage experiment through the murine kidney of using a low infectious dose. We found that the overall virulence of the commonly used wild type strain SC5314 did not change after eight passages and that the isolated pools showed only very moderate changes of phenotypic traits on the population level. Nevertheless, the last passage showed a higher phenotypic variability and a few individual strains exhibited phenotypic alterations suggesting that microevolution has occurred. However, the majority of the tested single strains were phenotypically indistinguishable from SC5314. Thus, our findings indicate that characteristics of SC5314 which are important to establish and maintain kidney infection over a prolonged time are already well developed.

## Introduction

The diploid fungus *Candida albicans* lives as a harmless commensal on mucosal surfaces of the gastrointestinal and reproductive tracts of most healthy humans. However, *C. albicans* also has the potential to cause disease, ranging from superficial infections, such as oropharyngeal candidiasis, to life-threatening disseminated infections affecting various internal organs (invasive candidiasis). Risk factors for invasive candidiasis include antibiotic treatment, gastrointestinal surgery, indwelling catheters and other medical devices, and prolonged stay in an ICU [1]. The incidence of invasive candidiasis has remained unchanged or even increased during the last decades [2]–[4]. Despite the use of modern antifungals the overall mortality is still high [5], [6]. Thus, invasive candidiasis presents a persistent problem in patients at risk [2], [3].

Development of clinical disease depends on both host susceptibility, like the immune status, and *C. albicans* virulence traits [7]–[9]. Another prerequisite is the ability of *C. albicans* to adapt to different host niches during the infection process [10], [11]. This adaptation is mediated short-term by changes in gene expression, translation and post-translational modifications. Additionally, genetic alterations like nucleotide exchanges, insertions, deletions, chromosomal rearrangements and copy-number variations of chromosome segments or whole chromosomes can lead to phenotypic variation within *Candida* populations [12], [13]. The gradual development of phenotypic variants by genetic modifications under selection pressure is called microevolution [14]–[16]. Microevolution of *C. albicans* has been experimentally confirmed as a mechanism of adaptation to antifungal drugs *in vitro* [17], [18] and has also been identified to be involved in the development of drug resistance *in vivo* [19]–[21]. Furthermore, microevolution of *C. albicans* occurs in the commensal state during long-term colonization of the human gastrointestinal tract as well as in recurrent vaginal infections [14], [22]. The latter suggests that microevolution may be important during *C. albicans* infections. Supporting this hypothesis, Forche *et al.* showed that a single passage through a mouse host leads to variations in colony growth and morphology associated with long-range loss of heterozygosity and chromosome rearrangement events [23]. However, to our knowledge the consequences of microevolution for virulence have yet only been addressed in one study: Cheng *et al*. repeatedly transferred *C. albicans* isolated from the spleen of infected mice to the blood stream of healthy animals [24]. The fifth passage resulted in a stable respiration-deficient isolate displaying delayed filamentation initiation and abnormalities in carbon-assimilation, thus further supporting the concept of microevolution during *C. albicans* infection [24].

The main target organ in murine *Candida*-sepsis is the kidney [25], [26]. In contrast to the spleen, the fungal burden in the kidneys increases over time, leading to a strong proinflammatory response without clearing infection [27]. To determine whether microevolution plays a role in establishment of infection and fungal persistence within the kidney, we performed a long-term serial passage experiment in which mice were systemically infected with a low dose of *C. albicans* cells isolated from the kidneys of mice 14 days after infection. The results of this serial passage experiment demonstrate that passaging through the kidney leads to increased phenotypic variability within the fungal population, possibly by microevolution. However, the overall virulence and fungal fitness, as well as the host response, varied between infected animals without a clear trend over the passages, suggesting that the *C. albicans* strain used, SC5314, is well adapted to infect and persist in the murine kidney.

## Materials and Methods

### Ethics Statement

All animal experiments were in compliance with the German animal protection law and were approved by the responsible Federal State authority (Thüringer Landesamt für Lebensmittelsicherheit und Verbraucherschutz) and ethics committee (beratende Komission nach § 15 Abs. 1 Tierschutzgesetz; permit no. 03–006/09).

### Strains and Culture Conditions

*C. albicans* SC5314 [28] was used for initiation of the serial infection experiment. Colonies isolated from infected mouse kidneys (passage 1 to 8) were obtained as described below. All strains were maintained as glycerol stocks at −80°C. Individual strains were obtained by plating either SC5314 or kidney isolates from the glycerol stock on YPD agar plates (1% w/v peptone, 1% w/v yeast extract, 2% w/v glucose, 2% w/v agar). After two days incubation at 30°C, 40 colonies from each plate were selected and transferred to a 96 well plate (TPP) containing YPD medium. The plate was incubated at 30°C and 180 rpm for 24 h and subsequently used to prepare two glycerol stock plates. For all experiments, over night cultures were prepared by inoculating liquid YPD with glycerol stock culture followed by incubation at 30°C and 180 rpm.

### Preparation of Infection Inoculum

SC5314 maintained as glycerol stock at −80°C was streaked on a YPD agar plate and incubated for 48 h at 30°C. A single colony was used for subculture on YPD agar for 12 h at 30°C. From this subculture, a single colony was inoculated in liquid YPD and grown for 24 h at 30°C and 180 rpm. SC5314 cells were washed twice with ice-cold phosphate-buffered saline (PBS) and adjusted to 1×10^6^/ml in PBS. The infection dose was confirmed by plating serial dilutions of the infection suspension on YPD plates. For subsequent passages, *C. albicans* cells isolated from infected kidneys were used. Therefore, serial dilutions of kidney homogenate were plated onto YPD plates containing 50 µg/ml chloramphenicol and incubated for two days at 30°C. Colonies were swabbed from plates using a sterile cotton bud and resuspended in liquid YPD. In case both mice survived the infection, colonies recovered from the kidneys of both mice were used. After 24 h at 30°C and 180 rpm, *C. albicans* cells were harvested and prepared for infection as described for SC5314. An aliquot of the infection suspension was mixed with 85% glycerol and stored at −80°C (pool isolates). In total, eight rounds of infection were performed.

### Mouse Infection Model and Microevolution

Female BALB/c mice five to six weeks old (18–20 g; Charles River) were used for the microevolution experiment. The animals were housed in groups of two in individually ventilated cages and cared for in strict accordance with the principles outlined in the *European Convention for the Protection of Vertebrate Animals Used for Experimental and Other Scientific Purposes* (http://conventions.coe.int/Treaty/en/Treaties/Html/123.htm).

Two mice per passage were challenged intravenously on day 0 with 5×10^3^ *C. albicans* cells per g body weight via the lateral tail vein. After infection, the health status of the mice was examined twice a day by a veterinarian and surface temperature and body weight were recorded daily. Mice showing signs of severe illness (isolation from the group, apathy, >25% weight loss, hypothermia) were humanely sacrificed. Surviving mice were humanely sacrificed on day 14. Immediately after euthanasia, kidneys, spleen, liver and brain were removed aseptically, rinsed with sterile PBS, weighed, and kept in ice-cold lysis buffer (200 mM NaCl, 5 mM EDTA, 10 mM Tris pH 7.4, 10% glycerin, 1 mM phenylmethylsulfonyl fluoride, 1 µg/ml leupeptid, 28 µg/ml aprotinin) on ice. The organs were aseptically homogenized using an Ika T10 basic Ultra-Turrax homogenizer (Ika). The fungal burden was determined by plating serial dilutions of the homogenates on YPD plates containing 50 µg/ml chloramphenicol.

### Quantification of Myeloperoxidase (MPO) and Cytokines from Tissue Homogenates

For quantification of MPO and cytokines, tissue homogenates were centrifuged at 1.500 g, 4°C for 15 min. The first supernatants were centrifuged again and the obtained final supernatants were stored at −80°C. The MPO levels were determined using the MPO ELISA Kit (Hycult Biotechnology). For the quantification of IL-6 and GM-CSF, Mouse ELISA Ready-SET-Go (eBioscience) were applied according to the manufacturers instructions.

### Growth Rate Determination and Stress Resistance

Over night cultures of the individual strains were diluted 1∶15 in YPD or SD minimal medium (2% dextrose, 0.17% yeast nitrogen base, 0.5% ammonium sulfate) in a 96 well plate (TPP) and incubated at 30°C and 180 rpm. After 4 h the OD_600_ was determined and the strains were diluted in fresh media (YPD, SD and SD supplemented with 2 mM H_2_O_2_, respectively) to an optical density of 0.05. Cell growth was monitored over 24 h or 48 h in 96 well plates at OD_600_ at indicated temperatures using an Infinite M200 pro ELISA reader (Tecan). Measurements were performed every 30 min directly after a 5 sec shaking period (3 mm amplitude). Generation time of exponentially growing yeast was calculated by using the following formula: g = 1/[(lb_OD600 end_ – lb_OD 600 start_)/(t_end_ –t_start_)] (lb = binary logarithm, t = time). For stress resistance tests on solid media, over night cultures of the pools were washed twice with PBS and diluted to 2×10^3^ in PBS. 50 µl each were spotted onto SD agar (control) and on SD agar containing either 2 mM H_2_O_2_ (AppliChem), 1 µg/ml caspofungin (Merck & Co), 350 µg/ml Congo Red (Sigma) or 1.5 M sodium chloride (NaCl, Roth). Plates were incubated for 48 h at 30°C (H_2_O_2_, Congo Red, NaCl, caspofungin) or 42°C (temperature stress) and colony forming units (CFU) were determined. Survival was calculated by dividing the number of CFU on the stress plate by the number of CFU on the control plate. Experiments were performed in biological triplicates.

### Determination of Filamentation

To determine serum-induced filamentation in liquid media, overnight cultures were inoculated to Dulbecco Modified Eagles Medium (DMEM, PAA) containing 2 mM L-glutamine (PAA) and 10% heat inactivated fetal bovine serum (FBS, PAA) in 24 well plates (TPP) at a density of 1×10^6^ cells per well (germ tube formation) or 1×10^5^ cells per well (filament length), respectively. Plates were centrifuged (3 min, 500 g) to settle cells and then incubated at 37°C in the presence of 5% CO_2_ for 1 h and 4 h, respectively. Each experiment was performed in biological duplicates with two technical replicates. From each sample, germ tube formation was determined for 300 cells using an inverse microscope (Leica DMIL); the filament length of 40 cells per sample was measured using the inverse microscope and the software LAS (Leica Application Suite).

Filamentation under embedded conditions was determined for colonies grown in YPS agar (1% w/v yeast extract, 2% w/v bactopeptone, 2% w/v D(+)-saccharose, 2% w/v agar) incubated at 25°C for five days in biological triplicates. Colonies were categorized as follows: Category 1: low filamentation, zero to five filaments; category 2: moderately filamented, >5 filaments, that were shorter and less dense compared to category 3; category 3: highly filamented, >5 long filaments, which appeared as a very dense network.

Furthermore, hyphal formation was investigated on solid YPD agar supplemented with 10% FBS and on solid Spider medium (1% w/v mannitol, 1% w/v nutrient broth, 0.2% w/v K_2_HPO_4_, 1% w/v agar, pH 7.2, [29]). Serum agar plates were incubated for four days and Spider agar plates for seven days at 37°C.

### Macrophage and Oral Epithelial Cells

The human buccal carcinoma epithelial cell line TR-146 (Cancer Research Technology) [30] and the peritoneal macrophage-like cell line J774.A1 (Deutsche Sammlung von Mikroorganismen und Zellkulturen (DSMZ)) were cultured and passaged in DMEM supplemented with 10% heat inactivated FBS at 37°C and 5% CO_2_.

### Quantification of Damage to Host Cells

Damage of macrophages and oral epithelial cells was determined by measuring the release of lactate dehydrogenase (LDH). 3×10^4^ TR146 cells per well and 4×10^4^ J774 cells per well were seeded in 96 well plates (TPP) and kept at 37°C with 5% CO_2_. After one day of incubation, cells were washed twice with PBS and infected with *C. albicans* at an MOI of 1∶1 in DMEM +1% FBS for 24 h at 37°C and 5% CO_2_. The following controls were included in the assay: (i) medium only control (MOC), (ii) low control (LC) of uninfected host cells and (iii) high control (HC) of uninfected host cells lysed with 0.5% Triton X-100 (Ferak) in DMEM +1% FBS ten minutes before measurement. For LDH quantification the Cytotoxicity Detection Kit (Roche Applied Science) was used according to the manufacturer’s protocol. To calculate the cell damage, the MOC and the LC values were subtracted from all sample values and damage was expressed as percentage of the HC. Each experiment was performed in biological duplicates.

### Invasion Assay

Invasion rates were determined as described previously [31]. Briefly, 3×10^5^ TR146 cells per well were seeded on 15 mm diameters glass coverslips in a 24 well plate (TPP) and grown to confluency. For infection, the monolayers were washed with PBS and 1×10^5^ *C. albicans* cells were added to the TR146 cells. After 3 h of incubation at 37°C and 5% CO_2_ the epithelial cells were washed once with PBS and fixed with 4% paraformaldehyde (Roth). Non-invading fungal cells and fungal cell parts outside of host cells were stained for 45 min with fluorescein-conjugated concanavalin A (Con A, Invitrogen) in the dark with gentle shaking (70 rpm). Then, the cells were washed twice with PBS and the TR146 cells were permeabilized by adding 0.5% Triton X-100 in PBS for 5 min. Finally, the cells were washed twice with PBS and all *C. albicans* cells were stained with calcofluor white (Fluorescent brightener 28, Sigma) for 15 min. After three intensive washing steps with water the coverslips were mounted on microscopy slides with mounting media. Stained cells were visualized with epifluorescence (Leica DM5500B, Leica DFC360 FX) using the appropriate filter sets for detection of Con A and calcofluor white. For each sample 100 cells were examined and the percentage of invading fungal cells was determined by dividing the number of (partially) internalized cells by the total number of cells. The experiment was performed on three separate occasions.

### Statistical Analysis and Definition of Outliers

GraphPad Prism, version 5.00 for Windows (GraphPad Software, San Diego, CA) was used to plot and to analyze the data. Data of the pools were compared to SC5314 by one-way analysis of variance (ANOVA) followed by Bonferronis’ multiple-comparison test. The data of the individual isolates are shown as scatter plot with line for mean and were analyzed with the nonparametric Mann-Whitney test.

For comparison of variation within SC5314 and passaged cells, mean and standard deviation were determined from the values obtained for all individual strains tested. Outliers were defined as strains with values above or below the whole population mean ±2×standard deviation, and color-coded for illustration. The F-test was used to compare the variances of the populations of the 40 strains from SC5314 and Pool 8.

## Results and Discussion

### Passaging through the Murine Kidney does not Affect Overall Virulence of *C. albicans* SC5314

Serial passage experiments can be used to study factors which are important for local adaptation and therefore for the survival in the host [32]. For instance, the continuous passage of *C. albicans* through the murine spleen originated a strain that was more resistant to killing by neutrophils and attenuated in a systemic mouse infection model [24]. However, the kidney is the main target organ in systemic *C. albicans* infection in mice, and infection of the spleen differs from the kidney in several aspects. First, the kidney is the only organ that exhibits continuously increasing fungal burden after intravenous infection, whereas *C. albicans* is at least partially cleared from the spleen [25]–[27], [33]. Secondly, neutrophil accumulation in the spleen occurs rapidly but transiently without affecting the organ architecture. In contrast, in the kidney the neutrophil accumulation is delayed but persistent and results in formation of abscesses [25]. Furthermore, *C. albicans* is able to form filaments in the kidney but not the spleen, likely due to the differences in the immune response [25].

Given these organ-specific differences, we decided to perform serial passages of *C. albicans* through the murine kidney, focusing on cells which survived in the kidneys for a prolonged time, to determine whether microevolution is involved in kidney infection and persistence. We hypothesized that persistence in the kidney might be linked to reduced virulence, possibly accompanied by a dampened immune response.

Therefore, mice were infected intravenously with a low dose of SC5314 wild type cells and monitored over a period of 14 days. *C. albicans* cells recovered from the kidneys of mice surviving this period were combined, representing passage pool 1 (P1). This pool was used for the next round of infection. We decided to use pools rather than single strains to avoid introduction of an artifical population bottleneck and to be able to assess whether strains with altered phenotypes would become dominant within the population. In total, eight passages were performed. Clinical examination, body weight, fungal burden, myeloperoxidase (MPO) concentration and levels of IL-6 and GM-CSF were measured to determine severity of clinical disease, fungal proliferation and immune response (Figure S1).

**Figure 1:**
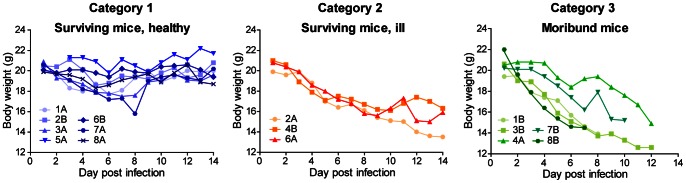
Body weight of individual mice during the *in vivo* microevolution experiment. Based on the health state mice were grouped into one of three categories: Category 1: Mice which survived the infection and appeared clinically healthy until day 14. Category 2: Mice that survived the infection but showed clinical symptoms of illness throughout the experiment. Category 3: Mice which became severely ill and had to be sacrificed before day 14. Lines show the body weight of individual mice, mice were designated as follows: 1. Number of passage; 2. Letter A or B (differentiating the two mice within each passage group). For example, the two mice used for the initial round of infection are designated as 1A and 1B, respectively. Mouse 5B died atypically before day 14 and is therefore not included in the figure.

**Figure 2:**
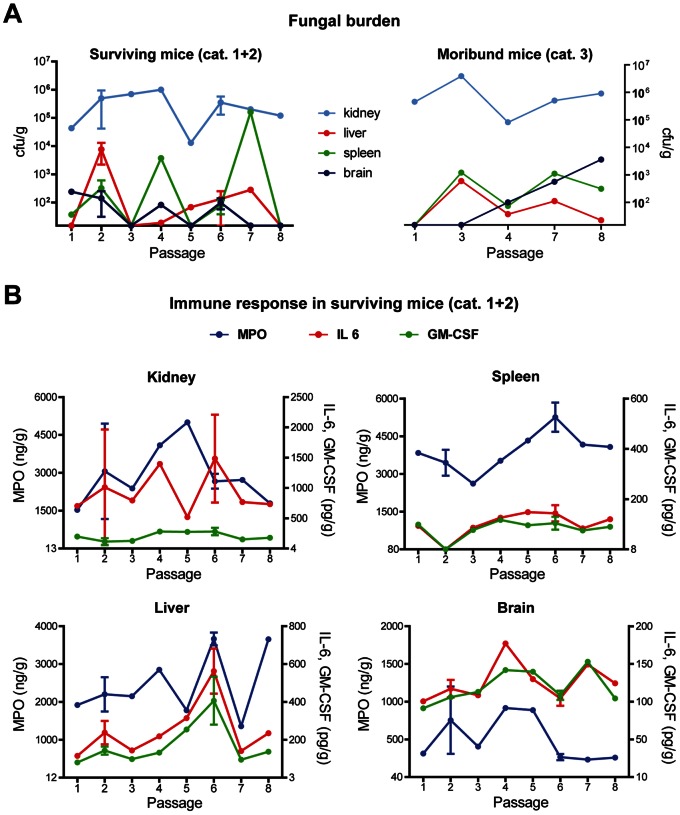
Fungal burden and immune response in systemically infected mice. (**A**) Fungal burden in kidneys (light blue), liver (red), spleen (green) and brain (dark blue) of systemically infected mice. The left graph shows data of mice surviving until the end of the experiment on day 14 (category (cat.) 1 and 2), the right graph shows fungal burden in moribund mice at the time of euthanasia. The X-axis is set as the passage number. (**B**) Evaluation of the immune response in different organs by quantification of MPO, IL-6 and GM-CSF by ELISA in tissue homogenates. The origin of the Y-axis is set to the detection limit. If both mice within a passage survived the infection, mean and standard deviation are presented.

As to be expected due to the low infectious dose [27], clinical symptoms within infected mice varied significantly (Fig. 1), ranging from mice which remained clinically healthy throughout the experiment or showed only mild, transient symptoms (category 1), over mice that showed clinical symptoms throughout the experiment but survived up to day 14 (category 2), to mice which became severely ill and had to be euthanized before the end of the experiment (category 3). The distribution of mice to the three categories was independent of the passage stage of the infection pool (Fig. 1). Consistent with published data [25], [33], the kidney was the only organ from which *C. albicans* could be readily isolated in high numbers 14 days after infection (Fig. 2A). This demonstrates the ability of *C. albicans* to persist in this organ, even in animals showing no clinical symptoms at the time of analysis. However, fungal burden in the kidney was independent of the passage. The fungal burdens of spleen, brain and liver were generally low and variable, without clear trend towards higher or lower fungal load over the passages.

In addition to fungal burden, the host response significantly influences the outcome of *C. albicans* infections [34], [35]. Therefore, the immune response was analyzed by determination of MPO, a marker for neutrophil infiltration [36], and the proinflammatory cytokines IL-6 and GM-CSF. IL-6 is associated with recruitment of neutrophils to the site of *C. albicans* infection in mice, and animals genetically deficient in IL-6 showed enhanced susceptibility to *C. albicans* [37], [38]. The production of GM-CSF is likewise induced upon *C. albicans* infection, and neutrophils activated with GM-CSF showed an enhanced phagocytosis rate and intracellular killing of *C. albicans* [39], [40]. The MPO concentrations in the kidney homogenates increased to a peak in passage 5 and decreased afterwards (Fig. 2B). The MPO levels in the other organs showed no specific pattern. IL-6 and GM-CSF were detected in all organs of surviving mice, except in the spleen of passage 2. The cytokine levels showed variations between animals but no clear passage-dependent trend. Thus, passaging *C. albicans* through the murine kidney did not significantly alter the renal immune response. In summary, the *in vivo* results indicated that virulence and pathogenesis remained unaltered after eight passages through the murine kidney.

### Passaging through the Murine Kidney Induced Moderate Phenotypical Alterations on the Population Level

**Figure 3:**
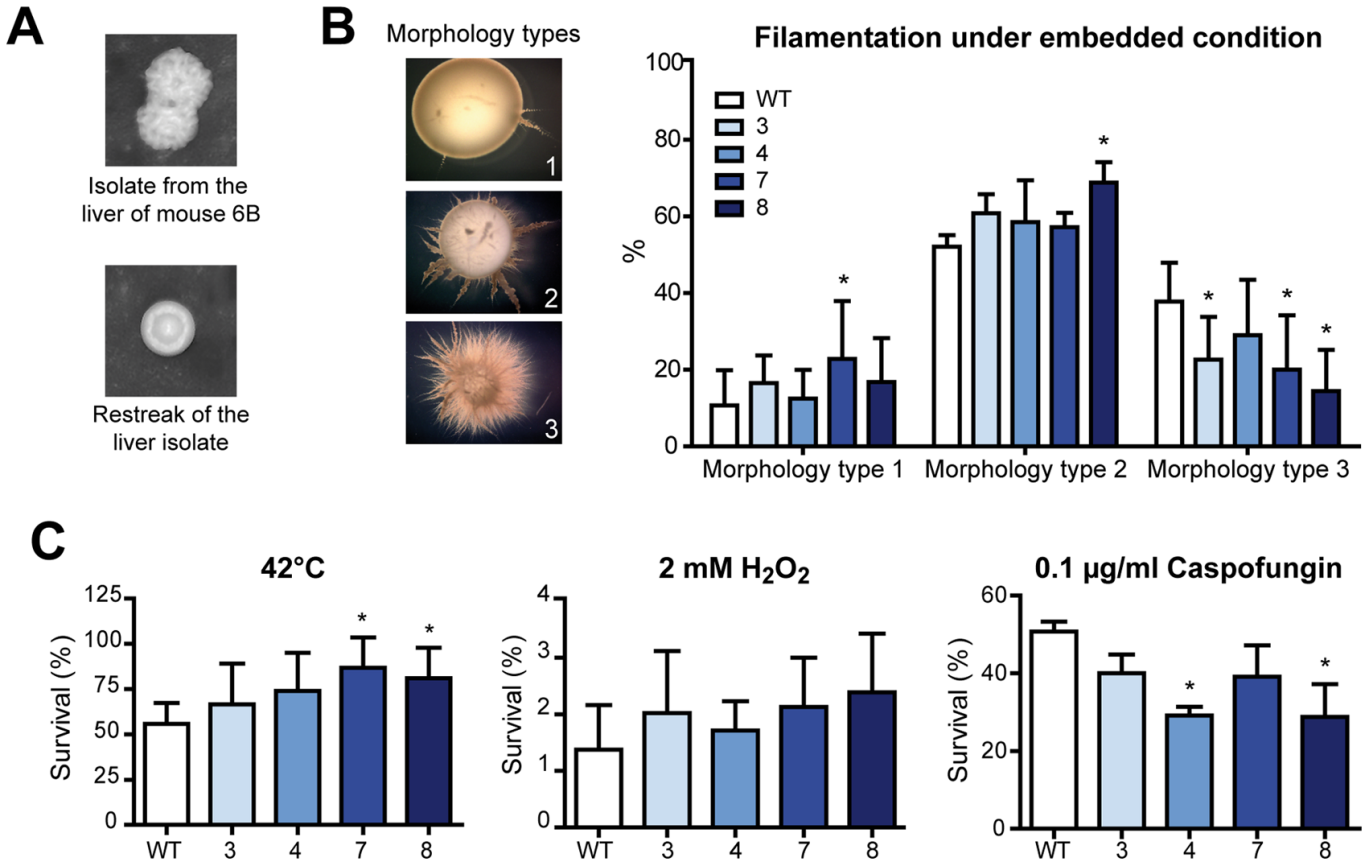
Phenotypical changes of passaged pools. (**A**) Clone displaying instable morphological alteration. (**B**) Filamentation under embedded conditions. Depending on the filamentation, colonies were placed in one of the following morphology types (examples depicted on the right side): Morphology type 1: reduced filamentation, zero to five filaments; morphology type 2: moderate filamentation, >5 filaments, that were shorter and less dense compared to morphology type 3; morphology type 3: strong filamentation, >5 long filaments, which appeared as a very dense network. The graph shows the distribution of colonies from each pool in the three morphology types. **(C)** Effect of different stresses in solid SD media on the survival of the pools. Survival was calculated by dividing the number of colonies on the stress plate by the number of colonies on a control plate. Error bars = standard deviation. * = p<0.05 compared to SC5314 (WT) by ANOVA followed by Bonferronis’s multiple-comparison test, n = 3.

It has been previously shown that *C. albicans* microevolution does occur in the murine kidney, even during a single passage, within 5–7 days [23]. In our experiment, colonies with altered morphology were isolated with a frequency of approx. 20% each from the liver of one mouse of passage 6 and from livers of both mice of passage 7. Albeit the phenotype was not stable upon subculture (Fig. 3A), the observation suggested adaptational changes in response to the host environment. We hypothesized that, although putative mutations were not sufficient to substantially change virulence on the population level, their impact on specific phenotypic traits might be detectable *in vitro*. Therefore, pools from selected passages were analyzed regarding filamentation, stress resistance and interaction with host cells.

Filamentation was determined in response to serum, in Spider medium and under embedded growth. The formation of germ tubes as well as the hyphal length in liquid serum-containing media (Figure S2A), hyphal formation on serum-containing agar and Spider agar (data not shown) did not significantly differ between the passaged pools. However, we observed differences under embedded growth, a condition that might simulate growth in tissue at least on the physical level [41]. Under these conditions, three out of four passaged pools showed significantly less colonies that exhibited a very dense filament network (category 3) as compared to SC5314 (Fig. 3B). This suggests a moderately impaired ability of passaged pools to form filaments under this specific condition.

Within the host, *C. albicans* is likely exposed to different stresses, especially high temperature and oxidative stress. Thus, we evaluated the resistance of passaged pools to thermal and oxidative stress as well as to osmotic stress, cell wall stress and the antifungal compound caspofungin. While resistance against cell wall stress induced by Congo Red and osmotic stress induced by NaCl (Figure S2B) was not altered in any of the pools, the passaged pools showed increased survival at 42°C, which was statistically significant for the two final passage pools P7 and P8. Resistance to oxidative stress (H_2_O_2_) did likewise increase but did not reach statistical significance (Fig. 3C). Additionally, P4 and P8 displayed lower resistance against caspofungin (Fig. 3C). Finally, we investigated whether the interaction with host cells differed between the pools. Consistent with the unaltered virulence *in vivo*, invasion into and cell damage of epithelial cells (Figure S2C) and the ability to damage macrophages (data not shown) were indistinguishable between passaged pools and SC5314.

In summary, these experiments showed that most phenotypic characteristics were retained within the pools over the passages. However, the increased tolerance to high temperature and oxidative stress, which represent stresses relevant *in vivo*, suggested moderate adaptation on the population level.

### Isolates from the Last Passage were Better Adapted to High Temperature and Showed a Higher Phenotypic Variability

**Figure 4:**
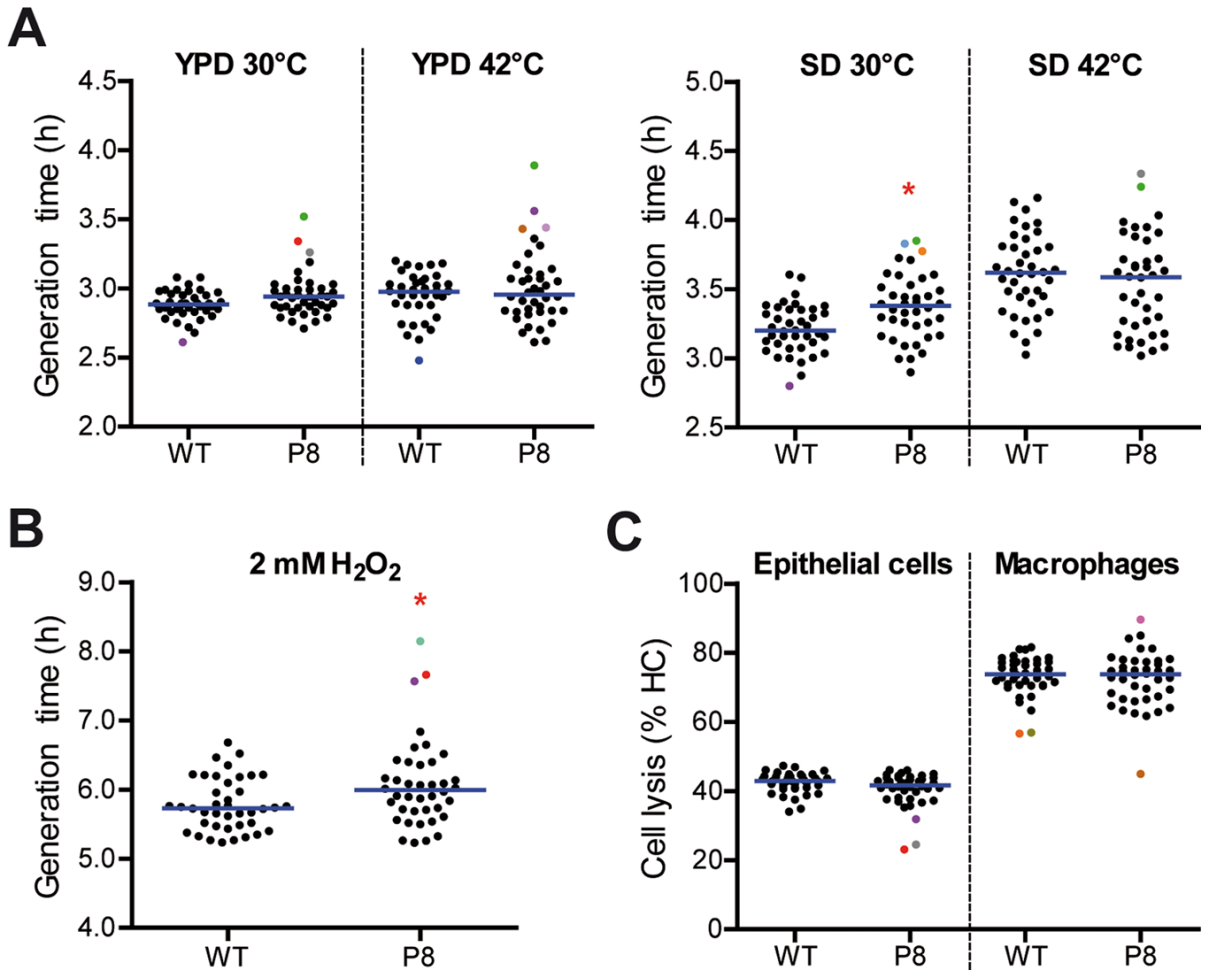
Characterization of single isolates from passage 8. (**A**) The graphs show the generation times of 40 strains of each WT and P8 at different media at 30°C and 42°C. (**B**) Generation times of 40 strains of each WT and P8 for H_2_O_2_ stress at 30°C. (**C**) Damage capacity of the 40 strains of each WT and P8 to epithelial cells and macrophages. WT = SC5314. P8 = passage 8. blue line = mean of the 40 strains. * = p<0.05 compared to WT by nonparametric Mann-Whitney test. n = 2 for growth experiments, and n = 3 for experiments with host cells, mean value is shown for each strain. Colored dots and triangles = outliers (for definition see material and methods), each color defines a specific strain.

Microevolution is a process which occurs in single cells within a population. Whether phenotypic alterations mediated by microevolution become evident on the population level depends on various factors, including the selective pressure and possible benefit of a mutation which determines the selective advantage of individual strains within populations. Thus, microevolution will only become phenotypically evident on the population level if the selective pressure favors mutants with certain properties. In the absence of a directed selective pressure, microevolution might occur within a population without changing the collective phenotype. A recent study showed that a single passage through the murine kidney is sufficient to increase population heterogeneity, both on the genome and phenotype level [23]. We assumed that, similarly, passaged pools from our experiment might have been enriched for strains with altered phenotypical properties. To test this hypothesis, we analyzed 40 randomly selected individual strains each from the last passage pool (P8) and SC5314 for growth speed, stress resistance and host cell interaction. Growth curves were generated for all 80 strains in complete (YPD) and minimal medium (SD) at different temperatures and to oxidative stress in minimal medium. In complete medium at either 30°C or 42°C, the mean growth rates of SC5314 and P8 strains were indistinguishable. However, when incubated in minimal medium at 30°C, mean generation times were significantly higher for the 40 strains from P8 (Fig. 4A), indicating slower growth at low temperatures. In contrast, SC5314 and P8 displayed indistinguishable mean generation times at 42°C in minimal medium (Fig. 4A). This result and the observation that P8 showed an increased survival at 42°C on population level (Fig. 3C) suggested that P8 strains are well adapted to elevated temperatures.

Because P8 showed moderately enhanced resistance to oxidative stress at the population level (Fig. 3C), we also determined the generation time of individual strains under oxidative stress. The mean doubling times of the individual strains of P8 under this stress were significantly higher in comparison to the SC5314 strains (Fig. 4B). However, the slower growth of P8 treated with H_2_O_2_ is likely due to the generally slower growth at 30°C. It should furthermore be noted that survival in the presence of H_2_O_2_ was increased at the population level for P8 (Fig. 3C), suggesting that slower growth and increased survival might be mechanistically linked. Finally, we tested the potential to damage epithelial cells and macrophages. The mean damage caused by P8 strains was similar to SC5314 (Fig. 4C), confirming the results obtained with pools.

In addition to mean growth and mean damage potential, these experiments also allowed us to estimate the phenotypic variability within the SC5314 stock and P8 pool. The higher variability within P8 was reflected by the higher standard deviations observed for growth, cell damage capacity and invasion into epithelial cells (Table 1). By applying the F-test, significant differences of the variances were identified for growth in complete medium at 30°C and 42°C, respectively, for growth under oxidative stress and for damage capacity. Interestingly, albeit mean growth rates and damage potential of P8 and SC5314 were similar, several individual strains of P8 displayed increased generation times in complex medium and minimal medium as well as under H_2_O_2_ stress (colored dots in Fig. 4A and B). Four P8 strains displayed slower growth in more than one condition. Similarly, individual strains with altered ability to damage host cells were identified in P8 (Fig. 4C).

**Table 1:** Mean and standard deviation of the 40 strains from WT (SC5314) and Pool 8, respectively, for various growth conditions and interactions with host cells.

	WT		Pool 8	
	Mean	Std. Deviation	Mean	Std. Deviation
**Without stress**				
YPD 30°C	2.9 h	0.10	2.9 h	0.16*
SD 30°C	3.2 h	0.18	3.4 h	0.24
**Thermal stress**				
YPD 42°C	2.9 h	0.16	3.0 h	0.27*
SD 42°C	3.6 h	0.29	3.5 h	0.35
**Oxidative stress**				
SD +2 mM H_2_O_2_	5.8 h	0.38	6.1 h	0.63*
**Interaction with host cells**				
Invasion (TR146)	20%	4.86	20%	6.20
Cell lysis (TR146)	43%	2.94	42%	5.04*
Cell lysis (J774)	73%	5.69	72%	7.91*

* = p<0.05 compared to WT by F-test.

Taken together, analysis of individual strains revealed that although the majority of P8 strains were phenotypically indistinguishable from SC5314, a few strains displayed phenotypic alterations, suggesting that microevolution occurred during the passages through the murine kidney. However, genotyping would be necessary to confirm that the observed differences in phenotype are indeed based on microevolution rather than stable epigenetic modifications. Phenotype alterations detected included both gain (survival at 42°C) and loss of fitness (growth rate, resistance to caspofungin) under the different conditions tested; this is consistent with results from evolution experiments with other organisms showing that an increased fitness for one condition is commonly associated with reduced fitness in other environments [42], [43].

Even though we observed increased phenotypic variability in passaged pools, the overall virulence of the passaged pools did not change. This outcome might be in part due to the experimental setup. To generate yeast cells suitable for infection, *in vitro* culture steps were necessary: First, plating on solid medium to remove host tissue, determine CFU and to remove and detect gross bacterial contamination. Secondly, colonies were propagated in liquid medium to obtain semi-synchronized cells in early stationary phase and yeast form only. Thus, *in vitro* culturing might have selected against mutant strains and favored strains which retained wild type-like abilities [44]. Furthermore, it should be noted that kidney infection *in vivo* is a two-stage process: Within one day after infection, *C. albicans* forms filaments which grow invasively in the renal cortex. Neutrophil recruitment within the first day is comparatively low [25]. Therefore, the initial stage of kidney infection is characterized by fungal filamentation, hyphal proliferation and tissue invasion. At the later stage of infection, *C. albicans* proliferates with hyphae extending to the renal tubules and pelvis while large numbers of neutrophils and macrophages are recruited [25]. Hence, the ability of *C. albicans* to withstand the immune response is likely a crucial feature allowing fungal cells to persist. As we isolated *C. albicans* 14 days after infection, the ability to persist determined the strains in the recovered pool; however, these strains would also need to have a sufficient ability for filamentation and invasion to establish infection in the next round of *in vivo* selection. It appears therefore likely that the sequential infections selected for an “all-rounder” phenotype and that SC5314 is already well adapted to both establishment of systemic infection and persistence within the kidney.

Interestingly, Forche *et al*. identified several strains with wrinkling morphology after a single renal passage [23]. In contrast, we did not observe stable hyperfilamentous strains within the re-isolated pools, and pools rather showed a moderately reduced capacity to filament under embedded conditions. We suggest that these differences might be the consequence of the experimental designs: First, we used a lower inoculum to facilitate prolonged survival of infected mice. Secondly, we re-isolated *C. albicans* from clinically healthy mice or animals with a chronic infection whereas the other study used kidneys from mice succumbing to acute lethal infection. We thus speculate that increased filamentation might provide a selective advantage in acute infection but not chronic persistence.

To our knowledge, there is only one additional study investigating the effects of serial passages on the phenotype of SC5314. Cheng *et al.* performed serial passaging through the spleen, resulting in a strain with reduced growth *in vitro*, strongly reduced virulence in murine systemic infection but increased resistance to phagocytosis and retained ability to persist in the kidney [24]. This contrasts our experiment of kidney passages which did not produce a pool with altered virulence. We suggest that this difference is likely due to the distinct organ environment. In contrast to the kidney, *C. albicans* SC5314 does not filament within the spleen and fungal burden in this organ decreases over time [25]. Thus, phagocyte exposure is likely a strong selective force throughout the whole course of infection in the spleen. Furthermore, the steady decline in the number of fungal cells in the spleen creates a population bottleneck which reduces the variation in the fungal population and thus makes it more likely that mutations which further survival become enriched.

In summary, repeated passages of SC5314 through the kidney of systemically infected mice did not induce alteration of virulence and resulted in only moderate changes of phenotypic traits on the population level. The phenotypic variation within the population suggests that microevolution events might have occurred; however, this assumption was not experimentally tested. We propose that the ability to establish and maintain kidney infection over a prolonged time requires complex abilities, which are already well developed in *C. albicans* SC5314.

## Supporting Information

Figure S1 **Experimental setup of the** ***in vivo*** **microevolution experiment.** Two BALB/c mice were challenged intravenously with SC5314 at an infectious doses of 5×10^3^ CFU/g body weight. After 14 days, kidney, brain, spleen and liver were removed aseptically for analyses of fungal burden, myeloperoxidase (MPO) and cytokine levels. Yeast colonies recovered from both kidneys were used for the next round of infection. Overall, eight serial passages of SC5314 through murine kidneys were performed. AB = antibiotic (chloramphenicol)
(TIF)

Figure S2 **Characterization of passaged pools.** (**A**) Germ tube formation after 1 h and hyphal length after 3 h in DMEM +10% serum at 37°C and 5% CO_2_, WT = SC5314. (**B**) Effect of cell wall stress (Congo Red) and osmotic stress (NaCl) in solid media on the survival of WT (SC5314) and the pools. Survival was calculated by dividing the number of colonies on the stress plate by the number of colonies on the control plate. (**C**) Invasion and damage capacity of WT (SC5314) and pools. Invasion was quantified after 3 h and damage after 24 h of co-incubation. Data are shown as mean+standard deviation.
(TIF)
