# Supplementary figures and images for: Serial Passaging of Candida albicans in Systemic Murine Infection Suggests That the Wild Type Strain SC5314 Is Well Adapted to the Murine Kidney
Source: PLoS One. 2013 May 30;8(5):e64482. doi: 10.1371/journal.pone.0064482 (PMC3667833; doi:10.1371/journal.pone.0064482)

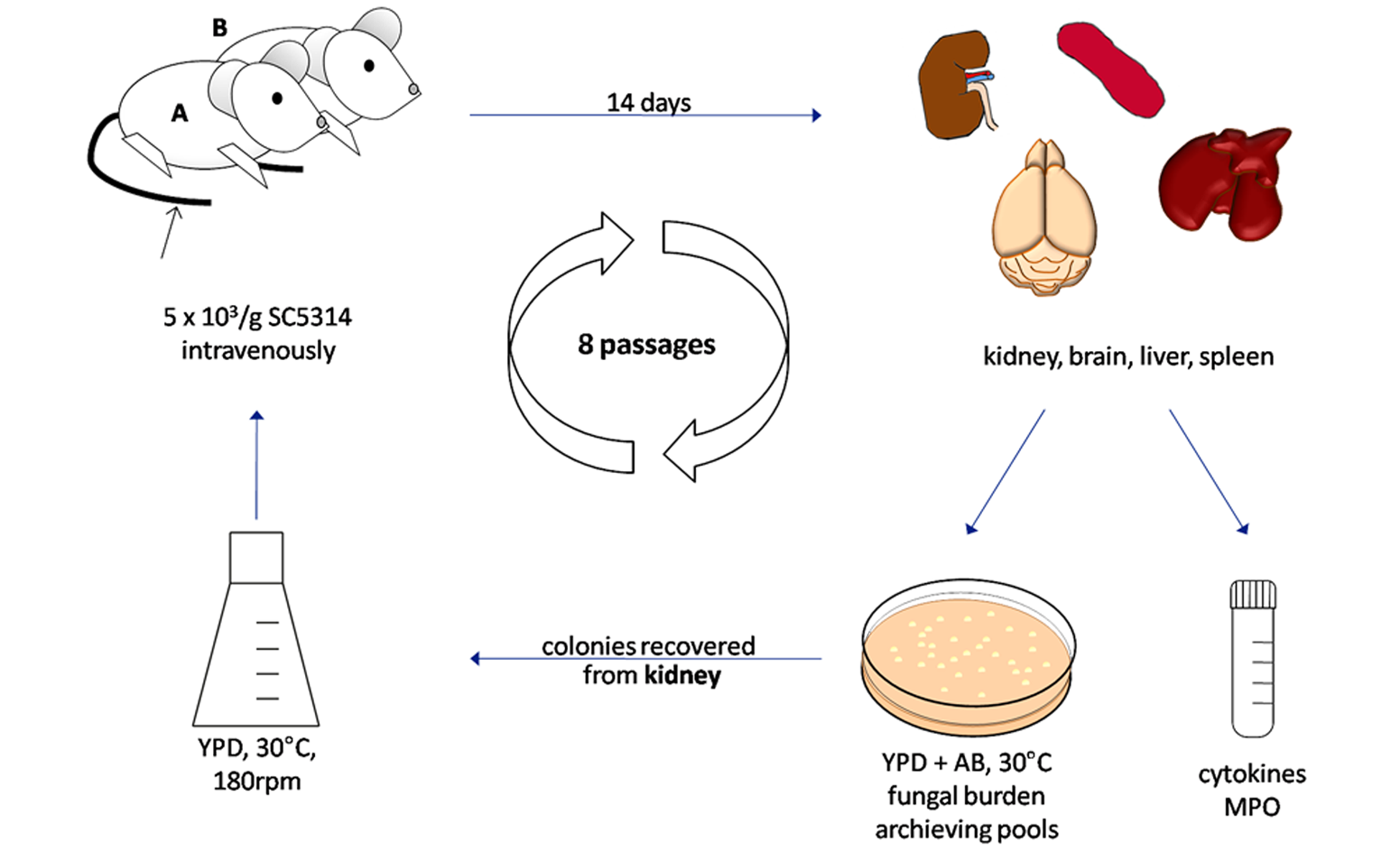

Supplement: Figure S1 — Experimental setup of the in vivo microevolution experiment. Two BALB/c mice were challenged intravenously with SC5314 at an infectious doses of 5×10^3^ CFU/g body weight. After 14 days, kidney, brain, spleen and liver were removed aseptically for analyses of fungal burden, myeloperoxidase (MPO) and cytokine levels. Yeast colonies recovered from both kidneys were used for the next round of infection. Overall, eight serial passages of SC5314 through murine kidneys were performed. AB = antibiotic (chloramphenicol) (TIF) [file pone.0064482.s001.tif]

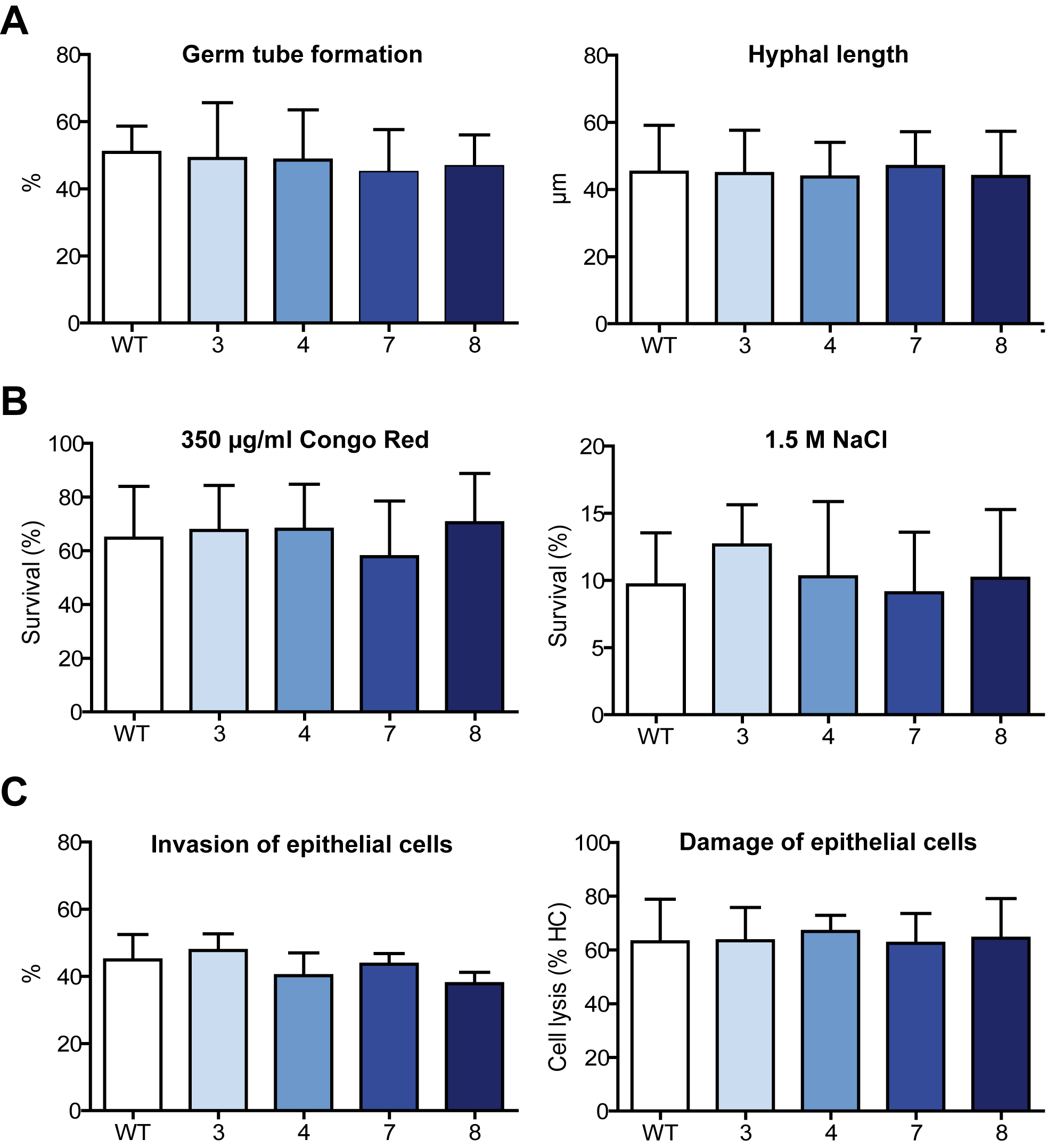

Supplement: Figure S2 — Characterization of passaged pools. (A) Germ tube formation after 1 h and hyphal length after 3 h in DMEM +10% serum at 37°C and 5% CO2, WT = SC5314. (B) Effect of cell wall stress (Congo Red) and osmotic stress (NaCl) in solid media on the survival of WT (SC5314) and the pools. Survival was calculated by dividing the number of colonies on the stress plate by the number of colonies on the control plate. (C) Invasion and damage capacity of WT (SC5314) and pools. Invasion was quantified after 3 h and damage after 24 h of co-incubation. Data are shown as mean+standard deviation. (TIF) [file pone.0064482.s002.tif]
